# The sensitivity of gamma-index method to the positioning errors of high-definition MLC in patient-specific VMAT QA for SBRT

**DOI:** 10.1186/1748-717X-9-167

**Published:** 2014-07-28

**Authors:** Jung-in Kim, So-Yeon Park, Hak Jae Kim, Jin Ho Kim, Sung-Joon Ye, Jong Min Park

**Affiliations:** 1 Department of Radiation Oncology, Seoul National University Hospital, Seoul, Korea; 2 Institute of Radiation Medicine, Seoul National University Medical Research Center, Seoul, Korea; 3 Biomedical Research Institute, Seoul National University College of Medicine, Seoul, Korea; 4 Center for Convergence Research on Robotics, Advance Institutes of Convergence Technology, Suwon, Korea; 5 Interdiciplinary Program in Radiation Applied Life Science, Seoul National University College of Medicine, Seoul, Korea; 6 Department of Radiation Oncology, Seoul National University College of Medicine, Seoul, Korea; 7 Program in Biomedical Radiation Sciences, Department of Transdisciplinary Studies, Seoul National University Graduate School of Convergence Science and Technology, Seoul, Korea

**Keywords:** Patient-specific quality assurance, Stereotactic body radiation therapy, Gamma-index method, Dose-volumetric indicator, High-definition multi-leaf collimator

## Abstract

**Background:**

To investigate the sensitivity of various gamma criteria used in the gamma-index method for patient-specific volumetric modulated arc therapy (VMAT) quality assurance (QA) for stereotactic body radiation therapy (SBRT) using a flattening filter free (FFF) photon beam.

**Methods:**

Three types of intentional misalignments were introduced to original high-definition multi-leaf collimator (HD-MLC) plans. The first type, referred to Class Out, involved the opening of each bank of leaves. The second type, Class In, involved the closing of each bank of leaves. The third type, Class Shift, involved the shifting of each bank of leaves towards the ground. Patient-specific QAs for the original and the modified plans were performed with MapCHECK2 and EBT2 films. The sensitivity of the gamma-index method using criteria of 1%/1 mm, 1.5%/1.5 mm, 1%/2 mm, 2%/1 mm and 2%/2 mm was investigated with absolute passing rates according to the magnitudes of MLCs misalignments. In addition, the changes in dose-volumetric indicators due to the magnitudes of MLC misalignments were investigated. The correlations between passing rates and the changes in dose-volumetric indicators were also investigated using Spearman’s rank correlation coefficient (γ).

**Results:**

The criterion of 2%/1 mm was able to detect Class Out and Class In MLC misalignments of 0.5 mm and Class Shift misalignments of 1 mm. The widely adopted clinical criterion of 2%/2 mm was not able to detect 0.5 mm MLC errors of the Class Out or Class In types, and also unable to detect 3 mm Class Shift errors. No correlations were observed between dose-volumetric changes and gamma passing rates (γ < 0.8).

**Conclusions:**

Gamma criterion of 2%/1 mm was found to be suitable as a tolerance level with passing rates of 90% and 80% for patient-specific VMAT QA for SBRT when using MapCHECK2 and EBT2 film, respectively.

## Background

A widely-adopted patient-specific quality assurance (QA) method for intensity modulated radiation therapy (IMRT) is the delivery of a verification plan, which is identical to the treatment plan, to a phantom loaded with a two-dimensional (2D) dosimeter. The measured 2D dose distribution is then compared to the distribution calculated by a treatment planning system (TPS) [1]. When comparing the 2D dose distributions, the gamma-index method suggested by Low *et al.* is generally used in the clinic and IMRT plans are evaluated with gamma passing rates [2]. This QA method for IMRT has also been applied to patient-specific QA for volumetric modulated arc therapy (VMAT). Although VMAT is different in many respects from IMRT, the criterion of 3%/3 mm has typically been used for gamma evaluation for both IMRT and VMAT QA [1,3-9]. Recent studies have raised the question of whether or not the criterion of 3%/3 mm for VMAT QA is clinically relevant [10-12]. Heilemann *et al.* recommended a stricter gamma criterion of 2%/2 mm rather than 3%/3 mm for a patient-specific VMAT QA [13]. Similarly, Fredh *et al.* compared VMAT QA results of 4 different commercial dosimeters and concluded that a criterion of 2%/2 mm rather than 3%/3 mm should be used clinically [14].

As previously mentioned, several studies have recently been performed that investigated patient-specific VMAT QA for conventional fractionated radiation therapy [14-16]. However, thus far no study has been performed to investigate VMAT QA results for stereotactic body radiation therapy (SBRT) in connection with high-definition MLC (HD-MLC) positioning errors in TrueBeam STx (version 1.6, Varian Medical Systems, Palo Alto, CA, USA) while using flattening filter free (FFF) mode. LoSasso *et al.* have demonstrated that dose error is inversely proportional to the mean MLC gap [17]. The same magnitudes of errors introduced in MLCs might affect the dose distribution of SBRT differently than that of conventional radiotherapy since an indication for SBRT is generally a small localized tumor [18]. Thus, the criterion of 2%/2 mm suggested by the recent studies for patient-specific VMAT QA is already widely-adopted for SBRT VMAT QA in many institutions [18].

In this study, we investigated the sensitivity of the gamma-index method with various gamma criteria by introducing intentional HD-MLC misalignments for SBRT in the FFF mode. The clinical relevance was evaluated by analysing changes in the dose volume histograms (DVH) of each structure.

## Methods

### Patient selection and simulation

After approval from an institutional review board (IRB, Seoul National University Hospital Human Research Protection Program Center), a total of 20 patients who underwent SBRT for either lung cancer (10 patients) or localized spine metastasis (10 patients) were selected for this study. All patients underwent CT scans using a Brilliance CT Big Bore™ (Philips, Amsterdam, Netherlands). The slice thicknesses of CT images for patients with lung cancer, and for localized spine metastasis were 2 mm and 1.5 mm, respectively. Four-dimensional CT images were acquired to allow delineation of internal target volume (ITV) for lung SBRT.

### Treatment planning of VMAT for SBRT

The VMAT plans for SBRT were generated using Eclipse™ (Varian Medical Systems, Palo Alto, USA) with 6 MV FFF photon beams for lung SBRT and 10 MV FFF photon beams for spine SBRT. TrueBeam STx with HD-MLC was used for the SBRT planning. Optimizations were performed with a progressive resolution optimizer 3 (PRO3, version A10) algorithm and dose distributions were calculated using the anisotropic analytic algorithm (AAA, version A10) with a calculation grid of 1 mm for the lung SBRT and 2 mm for the spine SBRT. The prescribed dose for lung SBRT was 48 Gy (12 Gy/fraction) with the exception of 2 cases where prescribed doses were 54 Gy and 60 Gy (13.5 Gy/fraction and 15 Gy/fraction). For spine SBRT, there were various prescribed doses ranging from 14 Gy to 30 Gy in 1 to 3 fractions. Single arc with full or partial rotation of the gantry was used for lung SBRT, while either single or two arcs were used for spine SBRT. The average value of monitor units (MUs) per cGy for the lung SBRT plans was 2.54, while that of the spine SBRT plans was 2.68. For lung SBRT plans at least 95% of the prescribed dose was delivered to 100% of the target volume, while for spine SBRT plans 100% of the prescribed dose was delivered to at least 90% of the target volume.

### Simulation of MLC misalignments in VMAT plans for SBRT

**Figure 1:**
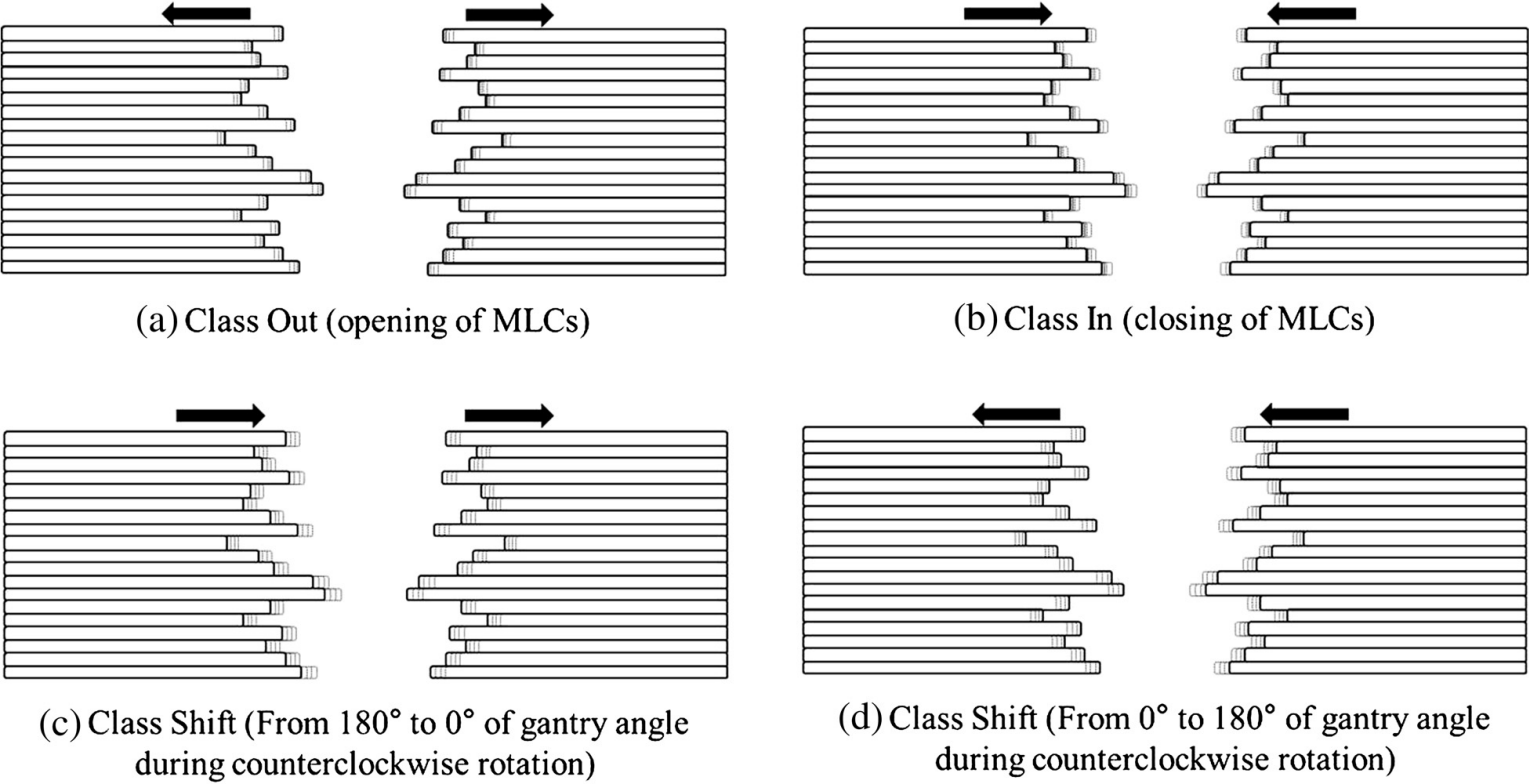
**Three types of MLC misalignments introduced to each SBRT plan delivered with VMAT technique.** The first type named as Class Out was a simulation that both multi-leaf collimators (MLCs) were opened **(a)** while the second type named as Class In was a simulation that both MLCs were closed **(b)**. The third type named as Class Shift was a simulation that both MLC banks were shifted in the same direction toward ground. MLC misalignments of Class Shift during gantry rotation from 180° to 0° **(c)** and from 0° to 180° **(d)** in the IEC scale are shown.

The original VMAT plans were exported from the TPS in DICOM format and then imported into an in-house program written in Matlab (version 8.1, Mathworks Inc., Natick, MA, USA), which allowed modification of the MLC movement. Three types of MLC misalignments were introduced to each VMAT plan (Figure 1). The first type, named *Class Out,* was a simulation where both MLCs were opened by 0.25 mm, 0.5 mm, 1 mm and 2 mm in an isoplane leading to larger openings of MLC apertures. The second type, *Class In,* was a simulation where both MLCs were closed by 0.25 mm, 0.5 mm, 1 mm and 2 mm leading to smaller openings of MLC apertures. The third type, *Class Shift*, was intended to simulate the effect of gravitational forces on MLCs. Both MLC banks were shifted in the same direction towards the ground with respect to the gantry angle by 1 mm, 2 mm and 3 mm. For each patient, 11 modified plans and 1 original plan were generated (12 plans per patient).

To eliminate the effect of the jaw tracking function of TrueBeam STx on dose distributions, the positions of the jaws were set to be identical for all the modified and original plans. Since the minimum dynamic leaf gap is fixed at 0.5 mm by the manufacturer, if the results of the modification for the *Class In* error were less than 0.5 mm, they were set to be 0.5 mm. Even though the manufacturer of the HD-MLC specifies that the positioning accuracy in terms of end accuracy is 1 mm, we introduced MLC errors of up to 2 mm for both *Class In* and *Class Out* because the value of dynamic leaf tolerance was set to be 2 mm in our institution, following recommendations from LoSasso *et al. *[17]. For *Class Shift*, MLC errors of up to 3 mm were simulated in order to consider not only the MLC positioning errors, but also the MLC bank positioning errors due to gravitational force. The modified plans were imported back to the TPS and dose distributions were calculated with each patient’s CT image sets using a calculation grid of 1 mm. The changes in DVHs of each structure in the modified plans were reviewed by radiation oncologists to determine if the changed DVHs were clinically acceptable or not.

### Dose distribution measurements of VMAT plans for SBRT

The planar dose distributions of each plan were measured with a MapCHECK2 detector array (Sun Nuclear Corporation, Melbourne, FL, USA) and EBT2 film (Ashland Inc., Covington, KY, USA). For gamma evaluations with MapCHECK2, CT images of the MapCHECK2 detector array inserted into a MapPHAN (Sun Nuclear Corporation, Melbourne, FL, USA) were acquired with a slice thickness of 1 mm. The reference dose distributions of each original VMAT plan were calculated using those CT images with a calculation grid of 1 mm which is the finest resolution in Eclipse™. Similarly, for gamma evaluations with EBT2 films, CT images of a solid water phantom (Gammex, Middleton, WI, USA) with a slice thickness of 1 mm were acquired and the reference dose distributions were calculated using those CT images with a calculation grid of 1 mm (30 cm × 30 cm × 10 cm) [2]. The original and modified VMAT plans were delivered by TrueBeam STx which was calibrated before delivery to keep the output deviation less than 0.1% following the AAPM TG-51 protocol [19]. The absolute dose and the responses of MapCHECK2 and EBT2 films were also calibrated before measurements. The size of the EBT2 film was 8 × 8 cm^2^ for the lung QA, and 12 × 12 cm^2^ for the spine QA. The EBT2 film was placed between two pieces of solid water phantom at 5 cm depth and the isocenter was located at the center of the phantom. Film dosimetry carefully followed the self-developing procedure recommended by manufacturer. Three separate batches of EBT2 film were used for the measurements to avoid the inter-batch response variation of EBT2 films. The films from each batch-numbered packet were used for each calibration. The EBT2 films were scanned 20 hours after irradiation using a flatbed scanner (Epson 10000XL, Epson Canada Ltd., Toronto, Ontario, Canada) in 48 bit color mode (*i.e.*, RGB mode). The practical spatial resolution of the scans was 75 dpi. The dual channel method for red and blue correction and scanner uniformity correction was applied for calibration. The calibration curve was acquired in the range of 0 to 26 Gy. The measured values of optical density were converted into a dose map using the RIT 113 film dosimetry system (version 6.0, Radiological Imaging Technology Inc., Colorado Springs, CO, USA). The calculated and measured dose distributions were also compared using the RIT 113 software. The calculated dose distributions were registered to the measured dose distributions with best fit method. For each patient a rectangular region of interest (ROI) was defined which covered not only the target area, but also the area below 10% of the maximum dose.

In the case of MapCHECK2, SNC patient software (version 6.1.2, Sun Nuclear Corporation, Melbourne, FL, USA) was used for analysis of the measurements. The calculated dose distributions with TPS were registered to the measured dose distributions by matching isocenter of the calculated dose distributions to the origin of the measured dose distributions.

### Data analysis

The absolute gamma analyses were performed using measurements from both MapCHECK2 and EBT2 films. The global gamma-index was used and the points with doses less than 10% of the maximum dose were ignored to reduce the effect of noise [20,21]. The gamma criteria of 1%/1 mm, 1.5%/1.5 mm, 1%/2 mm, 2%/1 mm and 2%/2 mm were used and the passing rates of each criterion vs. magnitudes of MLC misalignments were evaluated. Since various gamma criteria were tested in the present study, the passing rate was fixed to allow for direct comparisons to be made. The tolerance level of the passing rate was set to be 90% which is used in our institution as well as generally cited in the literature [22]. Linear regressions were used to find the lines of best fit for the results of the gamma passing rates vs. MLC misalignments. The gradients of the lines of best fit were compared one another to evaluate the sensitivity of each gamma criterion. The DVHs of the original VMAT plans were compared to those of the modified VMAT plans to investigate the effects of MLC misalignment on dose distributions. The changes in DVHs according to both type and magnitude of MLC misalignments were each reviewed by a radiation oncologist whose specialty is lung and spine SBRT. Clinically tolerable magnitudes of MLC misalignments were then determined for each type of MLC misalignment. The correlations between gamma passing rates of each criterion and the dose-volumetric changes in the target volume were investigated using Spearman’s rank correlation coefficient (γ). With a passing rate of 90% as a tolerance level, the receiver operating characteristic (ROC) curves and the values of area under curve (AUC) were acquired for each tested gamma criterion.

## Results

### Passing rates of gamma-index method with various gamma criteria with respect to the magnitudes of MLC misalignments

**Figure 2:**
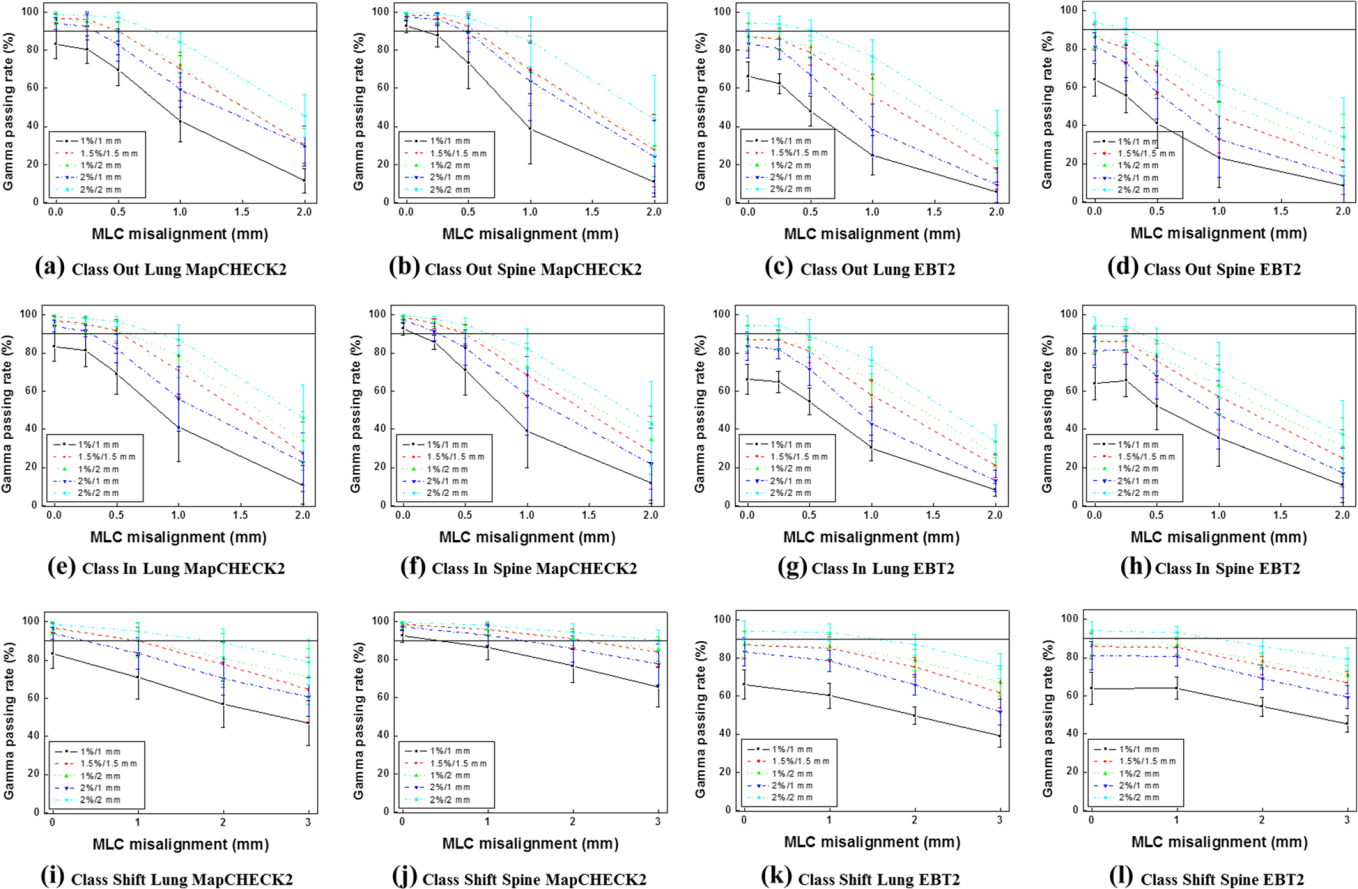
**Decreases of the absolute gamma passing rates according to the magnitudes of MLC misalignments.** The results at Class Out, Class In and Class Shift using MapCHECK2 in lung stereotactic body radiation therapy (SBRT) plans are shown in **(a)**, **(e)** and **(i)** while those in spine plans are shown in **(b)**, **(f)** and **(j)**, respectively. The results at Class Out, Class In and Class Shift using EBT2 films in lung SBRT plans are shown in **(c)**, **(g)** and **(k)** while those in spine plans are shown in **(d)**, **(h)** and **(l)**, respectively.

The relationships between the absolute gamma passing rates vs. MLC misalignments of *Class Out*, *Class In* and *Class Shift* with various criteria using MapCHECK2 and EBT2 films are illustrated in Figure 2. The gradients acquired from the lines of best fit of the passing rates with respect to the magnitudes of MLC misalignments are shown in Table 1. Higher absolute values of the gradient indicate higher sensitivity of gamma evaluation, thus the most sensitive criterion from the MapCHECK2 results was 1%/1 mm, and 2%/1 mm from EBT2 film.

**Table 1:** Gradients of best-fitting lines by linear regression of the absolute passing rates vs. MLC misalignments

		**1%/1 mm**	**1.5%/1.5 mm**	**1%/2 mm**	**2%/1 mm**	**2%/2 mm**
**Class Out (mm/%)**						
MapCHECK2	Lung	-37.7	-29.8	-30.3	-32.4	-15.5
	Spine	-41.5	-16.0	-22.6	-27.8	-5.1
EBT2	Lung	-31.6	-36.5	-30.8	-39.5	-26.3
	Spine	-28.0	-34.2	-30.9	-35.4	-29.5
**Class In (mm/%)**						
MapCHECK2	Lung	-37.9	-26.5	-24.3	-33.1	-10.9
	Spine	-41.1	-24.4	-20.4	-35.4	-12.6
EBT2	Lung	-30.5	-36.2	-32.6	-38.0	-30.1
	Spine	-28.3	-31.4	-28.2	-33.6	-25.8
**Class Shift (mm/%)**						
MapCHECK2	Lung	-12.4	-9.6	-7.9	-11.3	-5.2
	Spine	-8.3	-3.9	-3.4	-5.9	-2.5
EBT2	Lung	-9.4	-8.4	-6.6	-11.0	-5.8
	Spine	-7.5	-7.1	-6.0	-8.3	-5.2

*Abbreviation*: *MLC* multi-leaf collimator A higher absolute gradient means the test criteria are more sensitive to detect MLC misalignments*.*

For the MapCHECK2 results, the passing rates of the original plan with the criterion of 1%/1 mm were less than 90%. If a passing rate of 80% was applied as a tolerance level for 1%/1 mm criterion, MLC misalignments of 0.5 mm or less for *Class In* and *Class Out*, and 2 mm or less for *Class Shift* were not detectable. On the other hand, 2%/1 mm, which was the second most sensitive criterion with MapCHECK2, was able to detect 0.5 mm MLC misalignments for *Class In* and *Class Ou*t, and 1 mm for *Class Shift* with a passing rate of 90%. Gamma evaluations with the criteria of 1.5%/1.5 mm and 1%/2 mm were not sensitive enough to detect MLC misalignment up to 0.5 mm for *Class In* and *Class Out,* or up to 1 mm for *Class Shift*. The criterion of 2%/2 mm was the least sensitive.

The passing rates of EBT2 films were much lower than those of MapCHECK2. With the exception of the 2%/2 mm criterion, no criterion satisfied the passing rate of 90%. Therefore, a new tolerance level lower than 90% should be established for use of EBT2 films for VMAT. The most sensitive gamma criterion with EBT2 film was 2%/1 mm and it was able to detect MLC misalignment of 0.5 mm for *Class In* and *Class Out,* and 1 mm for *Class shift* with a passing rate of 80% as a tolerance level, showing the same performance as 2%/1 mm in the MapCHECK2 results.

The sensitivities of the gamma-index method with MapCHECK2 were higher in the lung plans than in the spine plans at all gamma criteria except 1%/1 mm for *Class Out* and 1%/1 mm, 2%/1 mm and 2%/2 mm for *Class In*. In the case of EBT2 films, the sensitivities in lung plans were higher than those in the spine plans in all cases except 1%/2 mm and 2%/2 mm for *Class Out*. For *Class Shift*, the sensitivities in the lung plans were always higher than those in the spine plans at all criteria with both MapCHECK2 and EBT2 films.

### Dose-volumetric changes with respect to the magnitudes of MLC misalignments for lung SBRT plans

The percent differences of dose-volumetric indicators with respect to the magnitudes of MLC misalignments in lung SBRT for *Class Out*, *Class In* and *Class Shift* are shown in Table 2 [23]. Since the kind of OARs which should be considered was different for each SBRT plan according to the tumor location, the evaluated numbers of plans (*N*) were not always same. The percent difference was calculated as

$$\%\mathrm{diff}=100\times \frac{\mathrm{Values}\;\mathrm{of}\;\mathrm{Modified}\;\mathrm{plan}-\mathrm{Values}\;\mathrm{of}\;\mathrm{original}\;\mathrm{plan}}{\mathrm{Value}\;\mathrm{of}\;\mathrm{original}\;\mathrm{plan}}$$

**Table 2:** The changes of dose-volumetric indicators according to the magnitudes of MLC misalignments in lung SBRT plans

		**Percent differences compared to the original plan (%)**									
**MLC error (mm)**	**Class**	**Target**			**Organs at risk**						
		**V**_**90%**_	**D**_**95%**_	**Mean**	**Trachea Max**	**SC Max**	**Rib Max**	**Lung V**_**12.4Gy**_	**Lung V**_**11.6Gy**_	**Heart Max**	**Esophagus Max**
***N***		**10**	**10**	**10**	**7**	**10**	**9**	**10**	**10**	**10**	**10**
0.25	Out	1.2 ± 1.3	1.9 ± 0.9	1.5 ± 0.6	2.5 ± 1.5	1.7 ± 0.7	2.3 ± 1.0	16.7 ± 12.0	-1.1 ± 31.2	2.8 ± 1.2	2.4 ± 0.8
	In	-1.7 ± 1.5	-1.9 ± 0.9	-1.5 ± 0.6	-2.9 ± 1.7	-1.7 ± 0.6	-1.8 ± 1.0	-18.1 ± 15.7	-8.9 ± 4.4	-2.2 ± 1.0	-2.3 ± 0.8
0.5	Out	2.0 ± 2.4	3.8 ± 1.7	3.1 ± 1.3	5.4 ± 3.1	3.5 ± 1.1	4.4 ± 1.9	33.1 ± 22.8	9.3 ± 35.5	5.4 ± 2.2	4.8 ± 1.7
	In	-4.0 ± 3.1	-3.9 ± 1.6	-3.1 ± 1.2	-5.6 ± 3.0	-12.2 ± 27.4	-4.0 ± 1.9	-33.6 ± 25.3	-17.9 ± 8.8	-5.0 ± 1.8	-4.5 ± 1.5
1	Out	2.7 ± 3.4	6.6 ± 2.9	5.6 ± 2.3	9.1 ± 5.7	6.1 ± 1.6	7.6 ± 3.2	40.1 ± 56.1	21.3 ± 40.7	9.4 ± 4.0	7.9 ± 2.9
	In	-10.1 ± 6.4	-7.1 ± 2.8	-5.6 ± 2.2	-9.1 ± 5.1	-5.8 ± 1.3	-7.0 ± 3.2	-50.3 ± 29.6	-32.8 ± 16.8	-8.5 ± 3.1	-7.5 ± 2.8
	Shift	-0.4 ± 1.8	-1.3 ± 2.2	0.1 ± 0.6	-1.3 ± 4.1	0.4 ± 1.4	0.9 ± 1.5	1.3 ± 5.6	-0.5 ± 2.0	0.1 ± 2.0	-2.0 ± 6.0
2	Out	3.4 ± 4.6	12.3 ± 5.4	10.9 ± 4.5	17.8 ± 11.1	12.5 ± 3.0	15.8 ± 7.9	85.6 ± 86.9	49.8 ± 54.7	19.0 ± 8.1	15.6 ± 5.6
	In	-40.0 ± 19.2	-15.1 ± 5.6	-11.5 ± 4.4	-17.2 ± 9.0	-11.8 ± 2.7	-13.8 ± 6.3	-68.8 ± 35.1	-62.0 ± 30.6	-17.5 ± 6.5	-14.7 ± 4.9
	Shift	-2.4 ± 3.4	-3.0 ± 3.3	-0.1 ± 1.1	-2.1 ± 6.5	0.7 ± 2.6	2.5 ± 3.7	2.8 ± 9.5	-2.8 ± 5.5	0.1 ± 4.3	-0.7 ± 1.7
3	Shift	-6.9 ± 5.4	-6.8 ± 5.6	-1.1 ± 1.8	-3.1 ± 8.7	1.4 ± 4.4	5.1 ± 6.0	1.1 ± 12.8	-5.4 ± 7.0	0.4 ± 7.2	-1.2 ± 2.5

*Abbreviations:MLC* multi-leaf collimator, *SBRT* stereotactic body radiation therapy, *V*_*n%*_ the volume irradiated at least *n%* of prescribed dose, *Max* maximum dose, *D*_*n%*_ the dose received by the *n%* volume of the structure volume, *SC* spinal cord, *N* number of plans.

In the case of *Class Out*, the doses delivered to both the target and the OARs were larger than those of the original plan since the sizes of MLC apertures were increased. Compared to the original plan, with a 0.5 mm MLC misalignment the averaged value of the normal lung volume that was irradiated by a dose larger than 12.4 Gy and 11.6 Gy was increased by 33.1% and 9.3%, respectively. With a 0.5 mm MLC misalignment, the averaged value of the maximum dose delivered to the spinal cord and heart was increased by 3.5% and 5.4%, respectively. In the case of *Class In*, the dose delivered to both the target and the OARs were smaller than those of the original plan since the sizes of MLC apertures were reduced. By introducing MLC errors of 0.5 mm, the averaged values of V_90%_, D_95%_ and mean dose to target were decreased by 4%, 3.9% and 3.1%, respectively. In the case of *Class Shift*, the values of V_90%_ and D_95%_ for the target were decreased. By introducing MLC errors of 3 mm, the averaged values of V_90%_ and D_95%_ for the target were decreased by 6.9% and 6.8%, respectively and the averaged value of maximum dose delivered to the ribs was increased by 5.1%. A radiation oncologist whose specialty is lung SBRT comprehensively reviewed the changed DVHs and concluded that 0.25 mm MLC misalignment in the case of *Class In* and *Class Out*, and 1 mm misalignment for *Class Shift* were clinically tolerable.

### Dose-volumetric changes with respect to the magnitudes of MLC misalignments for spine SBRT plans

**Figure 3:**
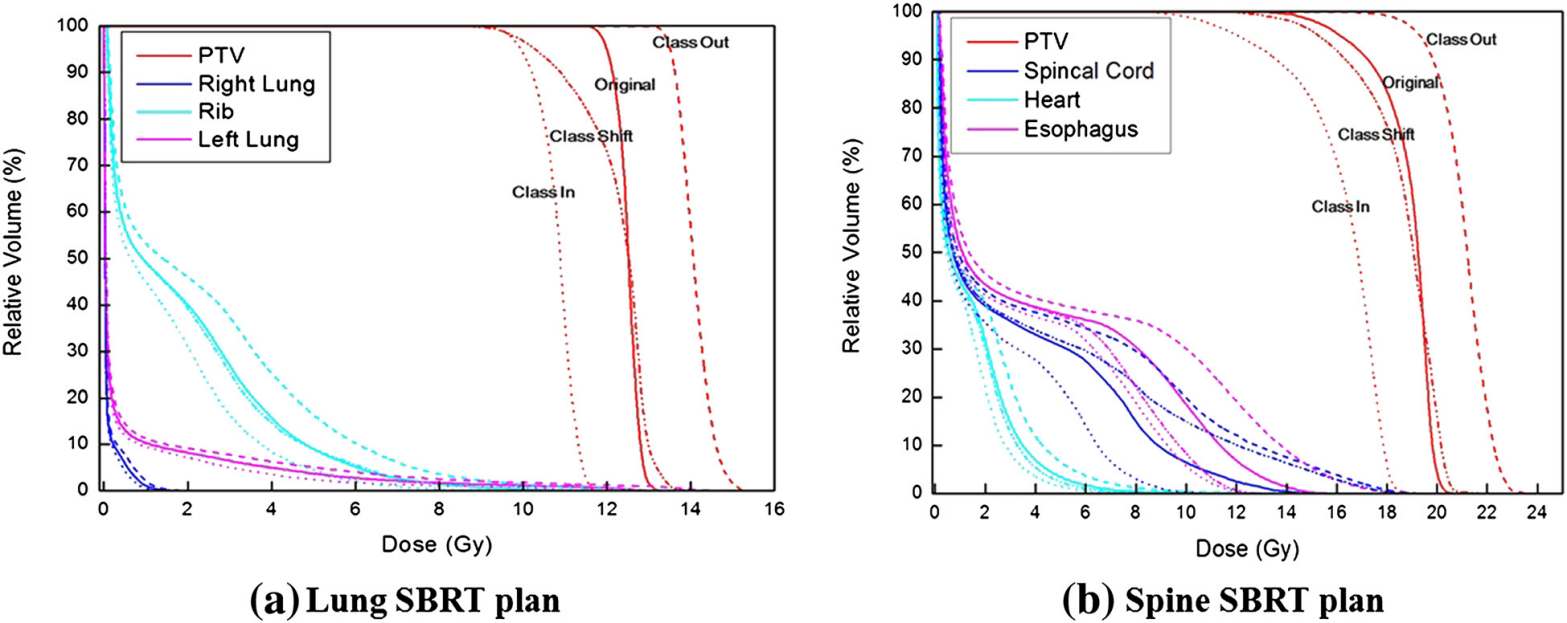
**Variation of dose volume histograms (DVH) due to the MLC misalignments.** Dose volume histograms (DVH) of a lung stereotactic body radiation therapy (SBRT) plan **(a)** and a spine SBRT plan **(b)** are shown. The DVHs from the original plan and the plans with multi-leaf collimator (MLC) misalignments of Class Out, Class In and Class Shift are shown. At Class Out and Class In, MLC misalignments of 2 mm were introduced while 3 mm errors were introduced at Class Shift.

The percent differences of dose-volumetric indicators with respect to the magnitudes of MLC misalignments in spine SBRT for *Class Out*, *Class In* and *Class Shift* are shown in Table 3. Since the kind of OARs which should be considered was different for each SBRT plan according to the tumor location, the evaluated numbers of plans (*N*) were not always same. In *Class Out*, by introducing MLC errors of 0.5 mm, the averaged value of V_10Gy_ of spinal cord was increased by 58.9% (*N = 3*). The averaged value of maximum dose delivered to the esophagus was increased by 10.3% (*N = 2*). In the case of *Class In*, with MLC errors of 0.5 mm, the averaged values of V_100%_ and D_95%_ of the target were decreased by 15% and 4.1%, respectively (*N = 8* and *N = 10*). In the case of *Class Shift*, the value of V_100%_ of the target was decreased by 5.4% (*N = 8*) when introducing MLC errors of 3 mm. With MLC errors of 1 mm, the averaged values of V_10Gy_ of spinal cord (*N = 3*), V_14Gy_ of cauda equina (*N = 1*) and the maximum dose to brain stem (*N = 1*) were increased by 36.8%, 123.9% and 11.8%, respectively. A radiation oncologist whose specialty is spine SBRT comprehensively reviewed the changed DVHs and concluded that 0.25 mm MLC misalignment in the case of *Class In* and *Class Out*, and 1 mm misalignment for *Class Shift* were clinically tolerable.An extreme example of the DVH changes of a lung and spine SBRT plan by the introduction of the maximum MLC misalignments in this study is shown in Figure 3.

**Table 3:** The changes of dose-volumetric indicators according to the magnitudes of MLC misalignments in spine SBRT plans

		**Percent differences compared to the original plan (%)**							
**MLC error (mm)**	**Class**	**Target**			**Organs at risk**				
		**V**_**100%**_	**D**_**95%**_	**Mean**	**SC V**_**10Gy**_	**Esophagus Max**	**Cauda equina V**_**14Gy**_	**Brainstem Max**	**Bowel Max**
***N***		**8**	**10**	**10**	**3**	**2**	**1**	**1**	**5**
0.25	Out	5.1 ± 2.2	2.4 ± 1.2	1.6 ± 0.7	28.5 ± 9.2	4.0 ± 1.0	133	7.4	1.4 ± 0.3
	In	-6.6 ± 4.8	-1.9 ± 1.2	-1.6 ± 0.7	-22.9 ± 3.7	-5.5 ± 3.2	-45.9	-1.9	-1.3 ± 0.4
0.5	Out	10.0 ± 5.1	4.5 ± 2.2	3.1 ± 1.4	58.9 ± 13.4	10.3 ± 5.3	356.3	12.1	2.7 ± 0.7
	In	-15.0 ± 7.7	-4.1 ± 2.3	-3.2 ± 1.4	-44.7 ± 6.8	-10.9 ± 7.0	-70.5	-7.4	-2.7 ± 0.7
1	Out	16.7 ± 9.3	7.2 ± 3.6	5.6 ± 2.5	113.9 ± 28.3	20.5 ± 12.2	610.6	19.5	4.8 ± 1.3
	In	-32.2 ± 13.6	-7.7 ± 4.0	-5.8 ± 2.6	-71.7 ± 7.4	-19.0 ± 11.5	-91.8	-14.8	-4.8 ± 1.3
	Shift	0.2 ± 1.5	1.1 ± 1.2	0.2 ± 0.3	36.8 ± 18.7	-9.6 ± 11.3	123.9	11.8	-2.4 ± 1.5
2	Out	28.5 ± 18.2	13.3 ± 6.4	10.9 ± 5.0	244.4 ± 41.9	45.0 ± 30.0	910.4	36.8	10.3 ± 1.8
	In	-65.5 ± 22.5	-16.6 ± 8.3	-11.8 ± 5.2	-98 ± 1.9	-33.8 ± 18.0	-100	-30.1	-9.3 ± 2.5
	Shift	-2.3 ± 2.8	-0.2 ± 1.9	-0.1 ± 0.7	66.4 ± 16.3	-15.2 ± 16.9	346.1	19.9	-4.3 ± 1.9
3	Shift	-5.4 ± 5.3	-3.3 ± 3.7	-0.8 ± 1.1	109.3 ± 23.5	-19.6 ± 20.0	628.3	27.6	-5.3 ± 2.5

*Abbreviations:MLC* multi-leaf collimator, *SBRT* stereotactic body radiation therapy, *V*_*n%*_ the volume irradiated at least *n%* of prescribed dose, *SC* spinal cord, *Max* maximum dose, *D*_*n%*_ the dose received by the *n%* volume of the structure volume, *N* number of plans.

### Ability of gamma criteria to detect the dose-volumetric changes in lung SBRT plans

The gamma criteria of 1.5%/1.5 mm, 1%/2 mm and 2%/2 mm with 90% passing rate were unable to detect increases of 33.1% and 9.3% of extra lung volume irradiated to at least 12.4 Gy and 11.6 Gy, respectively. These criteria were also unable to detect an increase in the maximum delivered dose to the trachea, spinal cord and ribs by amounts of 5.4%, 3.5% and 4.4%, respectively. Furthermore, decreases in V_90%_ and D_95%_ for the target volume of 4% and 3.9% were not detected with those gamma criteria. On the other hand, the gamma criteria of 1%/1 mm and 2%/1 mm were able to detect the dose-volumetric changes previously mentioned.

### Ability of gamma criteria to detect the dose-volumetric changes in spine SBRT plans

The gamma criteria of 1.5%/1.5 mm, 1%/2 mm and 2%/2 mm with 90% passing rate were unable to detect increases of 58.9% and 356.3% in the V_10Gy_ of the spinal cord and the V_14Gy_ of the cauda equina, respectively. These criteria were also unable to detect an increase in the maximum delivered dose to the esophagus, brainstem and bowel by amounts of 10.3%, 12.1% and 2.7%, respectively. Furthermore, decreases in V_100%_ and D_95%_ for the target volume of 15% and 4.1% were not detected with those criteria. On the other hand, the gamma criteria of 1%/1 mm and 2%/1 mm were able to detect the dose-volumetric changes previously mentioned.

### Correlations between dose-volumetric changes and gamma passing rates

No correlations were observed between dose-volumetric changes and gamma passing rates using either MapCHECK2 or EBT2 films with every criterion in both lung and spine SBRT plans (always γ < 0.4).

### Sensitivity and specificity

**Figure 4:**
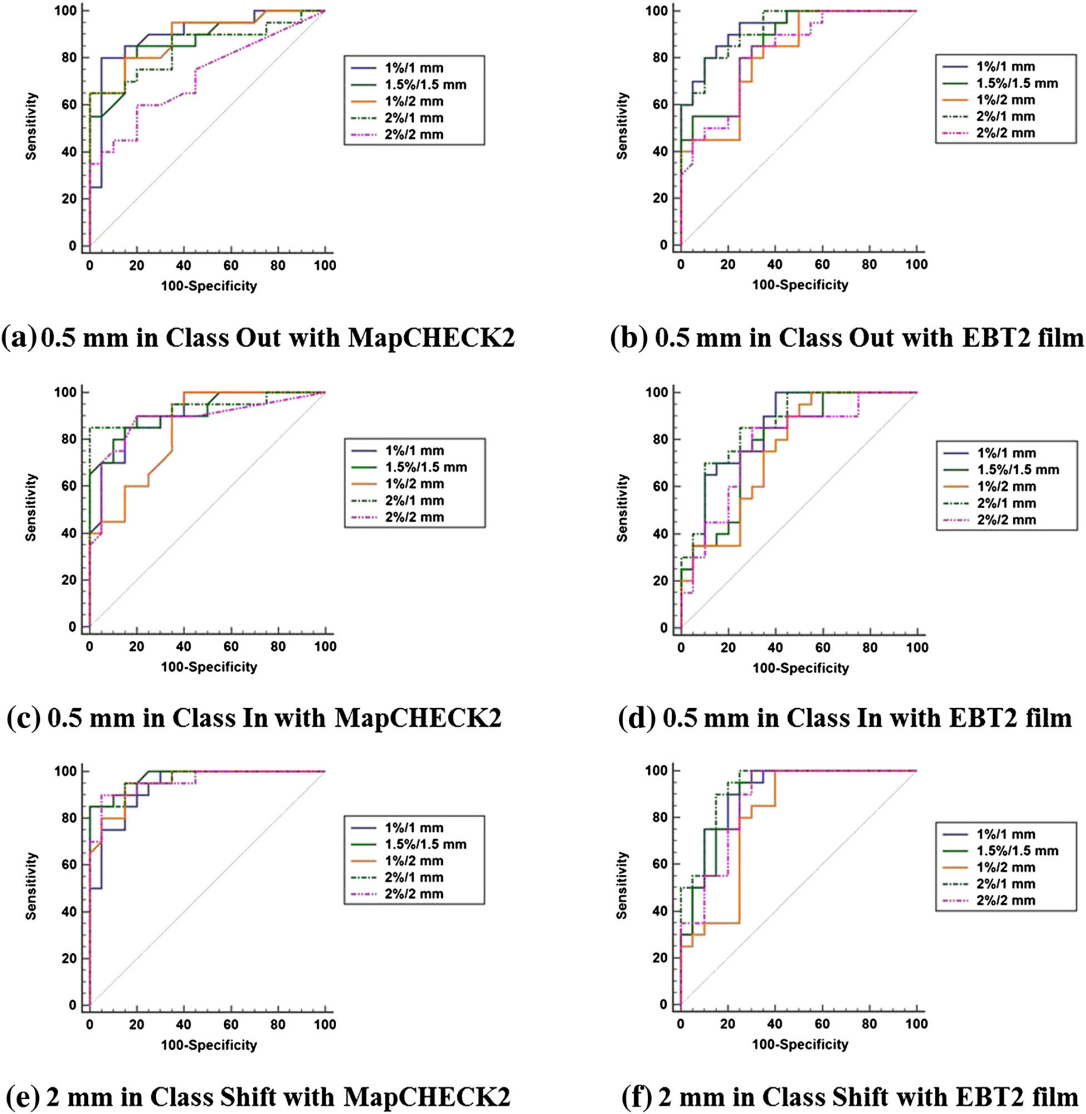
**Receiver operating characteristic curve of various gamma criteria when using MapCHECK2 and EBT2 film.** Receiver operating characteristic (ROC) curves of gamma criteria of 1%/1 mm, 1.5%/1.5 mm, 1%/2 mm, 2%/1 mm and 2%/2 mm are shown. The ROC curves of 0.5 mm MLC misalignment at Class Out **(a)**, Class In **(c)** and 2 mm MLC misalignment at Class Shift **(e)** when using MapCHECK2 are shown. Similarly, 0.5 mm MLC misalignment at Class Out **(b)**, Class In **(d)** and 2 mm MLC misalignment at Class Shift **(f)** when using EBT2 films are shown.

The ROC curves and the values of AUC are shown in Figure 4 and Table 4, respectively. Since we determined that clinically perturbed dose distributions appear from 0.5 mm MLC misalignments in *Class In* and *Class Out* and at 2 mm in *Class shift*, only those data are presented. In the case of MapCHECK2, 1%/1 mm showed the best performance in *Class Out,* and showed the worst performance in *Class Shift*. The gamma criterion of 1.5%/1.5 mm showed the best performance in *Class Shift*. The criterion of 2%/1 mm showed the best performance in *Class In* and the second best performance in *Class Shift*. In the case of EBT2 film, 2%/1 mm showed the best performance and 1%/1 mm showed the second best performance in *Class In* and *Class Shift*. For *Class Out*, 1%/1 mm showed the best performance and 2%/1 mm showed the second best performance.

**Table 4:** Area under curve (AUC) values for each gamma criterion when introducing 0.5 mm MLC misalignment in class in and class out and 2 mm MLC misalignment in class shift

	**MapCHECK2**			**EBT2 film**		
**Gamma criterion**	**Class In (0.5 mm)**	**Class Out (0.5 mm)**	**Class Shift (2 mm)**	**Class In (0.5 mm)**	**Class Out (0.5 mm)**	**Class Shift (2 mm)**
1%/1 mm	0.909	0.899	0.935	0.853	0.933	0.905
1.5%/1.5 mm	0.920	0.873	0.974	0.788	0.858	0.89
1%/2 mm	0.843	0.890	0.954	0.758	0.813	0.805
2%/1 mm	0.930	0.848	0.968	0.865	0.925	0.933
2%/2 mm	0.883	0.719	0.958	0.783	0.824	0.873

## Discussion

In this study, the gamma-index method criterion for SBRT VMAT plans that could achieve greater than 90% passing rates in original error free plans, while showing less than 90% passing rates in modified plans with MLC misalignments were investigated. Dose-volumetric changes were reviewed by a radiation oncologist from a clinical point of view and it was determined that *Class In* and *Class Out* modified plans with a 0.5 mm MLC misalignment, and *Class Shift* with a 2 mm misalignment were clinically unacceptable. In order to detect those MLC misalignments, the criterion of 2%/1 mm with 90% passing rate as a tolerance level seemed to satisfy the requirements mentioned above for MapCHECK2. The EBT2 films could satisfy the requirements when using gamma criterion of 2%/1 mm with a passing rate of 80%. The conventional 2%/2 mm criterion, widely-adopted in the clinic for SBRT plans, was not sensitive enough to detect MLC misalignments which may potentially impair the quality of treatment.

The most stringent criterion in this study, 1%/1 mm, seemed inappropriate since the original plan already failed, with passing rates less than the required 90%. In spite of the careful calibration of measuring devices as well as the institutional validation of TrueBeam STx and TPS before measurements, inherent uncertainties still existed in the original plans. Furthermore, setup uncertainties were also included in the results. Since these uncertainties could not be completely removed practically, when applying the criterion of 1%/1 mm, they may have influenced the gamma passing rates which were less than 90%. On the other hand, Poppe *et al*. demonstrated that statistical fluctuations and systematic errors are dominant when using 1%/1 mm criteria [24]. Similarly, Heilemann *et al.* didn’t recommend to use 1%/1 mm criteria [13]. Therefore, in order to use the criterion of 1%/1 mm, a careful approach is necessary and a different tolerance level should be established instead of 90%, as used in this study.

In the case of EBT2 film, in spite of excellent spatial resolution, the inherent uncertainty (approximately ±5%) is larger than the MapCHECK2 uncertainty [25,26]. The highest gamma criterion for dose used in this study was 1%, which was much lower than the uncertainty of EBT2 film. For this reason, the passing rates of the majority of the original plans were lower than 90% which is used as a tolerance level in the present study. In addition, the film to the calculated dose distribution registration in RIT113 program was done by fitting the gradients automatically resulting best match between the calculated and measured dose distributions. Therefore, 2D gamma analysis using EBT2 film was only an analysis of the dose distribution not of the overall accuracy of the system. However, the detecting ability of EBT2 film was found to be similar to MapCHECK2 as shown in Table 1. Moreover, gamma criterion of 2%/1 mm with a passing rate of 80% when using EBT2 film showed similar performance with that of 2%/1 mm with a passing rate of 90% when using MapCHECK2.

The sensitivities of gamma-index method to the MLC misalignments with various criteria were generally higher in the lung SBRT plans than spine SBRT plans. As previously studied by LoSasso *et al. *[17], since the lung target volumes were generally smaller than the spine target volumes, the sensitivity to the MLC misalignments was higher in the lung plans, in accordance with previous studies [13,17]. However, the magnitudes of the differences were smaller than those of previous studies since the differences in target volume between the lung and the spine SBRT plans relatively small compared to the previous studies [13,15].

Wagner and Vorwerk have suggested an increase of the tolerance level to up to 99% with conventional criterion instead of altering the criterion for the sensitive gamma-index method [9]. However, the results in this study show that increasing the tolerance level would not guarantee detection of small misalignments of the MLC. Similarly, Heilemann *et al.* demonstrated that the use of more stringent criterion rather than an increase of tolerance level would be beneficial [13].

No correlations between gamma passing rates and dose-volumetric changes were observed as previous studies have already shown in IMRT and VMAT cases [13,15,27,28]. Therefore, even though gamma-index is a convenient indicator for evaluation of patient-specific QA results, careful analysis of dose distributions should be completed by visual inspection by the physicist and clinician.

The results of the ROC curves and the values of AUC did not indicate a single gamma criterion as the ideal criterion in terms of sensitivity and specificity when clinically perturbed dose distributions began to appear in *Class In, Class Out and Class Shift* errors. The criterion of 2%/1 mm showed generally superior performance to the others when using both MapCHECK2 and EBT2 film although it was not always the best.

The results showed that small amounts of introduced MLC misalignments, even within the 1 mm specified by the manufacturer, hampered the plan quality severely. This is because the introduced MLC misalignments were systematic errors and not random errors. Entire MLCs were shifted in the present study, however this is not necessarily a realistic situation. Since the aim of this study is to investigate the ability of various gamma criteria to detect clinically unacceptable SBRT plans, and not to simulate real situations, we introduced systematic MLC misalignments which decreases plan quality.

In the present study, we adopted the global gamma-index method which is limited in its ability to review low dose regions. This limitation can potentially lead to misinterpretation of the delivered dose to OARs. Therefore, in the present study, radiation oncologists reviewed the changes in DVHs of modified plans and determined how much systematic MLC misalignment is clinically tolerable. As recommended by Heilemann *et al*., gamma passing rates are a good indicator for making a preliminary decision, however physicists have to evaluate the dose distribution carefully especially when using global gamma-index method.

One limitation of the present study is that the measured data were acquired with only MapCHECK2 and EBT2 films. The results in this study should not be directly applied to other patient-specific QA systems which use different measuring devices. However, we recommend that the gamma criterion of 2%/2 mm should be carefully examined, and alternatives considered for VMAT QA for SBRT.

Heilemann *et al.* and Fredh *et al.* have investigated appropriate criterion for 2D patient-specific QA in H&N, brain and prostate VMAT plans [13,14]. They have demonstrated that 2%/2 mm is a suitable criterion for fractionated VMAT QA. In this study, the appropriate criterion for the patient-specific VMAT QA for SBRT has been investigated [13,14]. The main difference between the present and previous studies is that we investigated specifically for SBRT QA using the VMAT technique. The SBRT plans should be delivered more accurately than conventional fractionated radiation therapy [18]. Therefore the gamma evaluation for SBRT requires more stringent criterion, however this criterion is not yet standardized. We recommend 2%/1 mm as an appropriate criterion for the gamma-index method for SBRT when using MapCHECK2 or EBT2 films.

## Conclusions

This study demonstrated that the widely clinically adopted gamma criterion of 2%/2 mm for patient-specific SBRT QA is not sensitive enough to evaluate the quality of VMAT plans for SBRT. When using the gamma-index method for SBRT VMAT QA, an appropriate gamma criterion for each QA system, which is able to detect errors that hamper plan quality should be established. It is recommended that 2%/1 mm be used as a criterion with tolerance levels for gamma passing rates of 90% and 80% when using MapCHECK2 and EBT2 films, respectively.

## Abbreviations

VMAT: Volumetric modulated arc therapy; SBRT: Stereotactic body radiation therapy; QA: Quality assurance; FFF: Flattening filter free; HD-MLC: High-definition multi-leaf collimator; IMRT: Intensity modulated radiation therapy; TPS: Treatment planning system; DVH: Dose volume histograms; ITV: Internal target volume; PRO3: Progressive resolution optimizer 3; AAA: Anisotropic analytic algorithm; MUs: Monitoring units.

## Competing interests

The authors declare that they have no competing interest.

## Authors’ contributions

JIK and JMP compiled and analysed dosimetric data and drafted the manuscript. SYP developed the in-house software to generate the DICOM plan with MLC errors. SJY oversaw its completion. JHK and HJK reviewed the modified treatment plans and determined whether those plans were clinically acceptable or not. JIK and JMP conceived of the study concept, participated in all aspects of its design and coordination and drafted the manuscript. All authors read and approved the final manuscript.
